# The characteristics of microbiome in the upper respiratory tract of COVID-19 patients

**DOI:** 10.1186/s12866-024-03281-w

**Published:** 2024-04-24

**Authors:** Xilong Zhang, Nadira Nurxat, Jueraiti Aili, Yakupu Yasen, Qichen Wang, Qian Liu

**Affiliations:** 1 Department of Laboratory Medicine, Ren Ji Hospital, Shanghai Jiao Tong University School of Medicine, 160 Pujian Road, Shanghai, 200127 China; 2 College of Health Science and Technology, Shanghai Jiao Tong University School of Medicine, Shanghai, 200025 China

**Keywords:** COVID-19, Co-infection, Upper respiratory tract, Microbiota, *Candida*

## Abstract

**Background:**

Co-infection with other pathogens in coronavirus disease 2019 (COVID-19) patients exacerbates disease severity and impacts patient prognosis. Clarifying the exact pathogens co-infected with severe acute respiratory syndrome coronavirus 2 (SARS-CoV-2) is premise of the precise treatment for COVID-19 patients.

**Methods:**

Sputum samples were collected from 17 patients in the COVID-19 positive group and 18 patients in the COVID-19 negative group. DNA extraction was performed to obtain the total DNA. Sequencing analysis using 16S and ITS rRNA gene was carried out to analyze the composition of bacterial and fungal communities. Meanwhile, all the samples were inoculated for culture.

**Results:**

We did not observe significant differences in bacterial composition between the COVID-19 positive and negative groups. However, a significantly higher abundance of *Candida albicans* was observed in the upper respiratory tract samples from the COVID-19 positive group compared to the COVID-19 negative group. Moreover, the *Candida albicans* strains isolated from COVID-19 positive group exhibited impaired secretion of aspartyl proteinases.

**Conclusion:**

COVID-19 positive patients demonstrate a notable increase in the abundance of *Candida albicans*, along with a decrease in the levels of aspartyl proteinases, indicating the alteration of microbiota composition of upper respiratory tract.

**Supplementary Information:**

The online version contains supplementary material available at 10.1186/s12866-024-03281-w.

## Introduction

Severe acute respiratory syndrome coronavirus 2 (SARS-CoV-2), as a novel member of enveloped RNA β-coronavirus, triggers coronavirus disease 2019 (COVID-19). The main symptom for COVID-19 includes severe SARS-CoV-2 associated pneumonia [1]. Since its emergence in 2019, COVID-19 has caused a global pandemic due to the rapid transmission [2]. The high mortality rate of COVID-19 poses a significant threat to human health and public health security. According to the statistics from the World Health Organization (WHO), as of July 12th, 2023, there have been a total of 760 million COVID-19 confirmed cases and 6.95 million deaths worldwide. Fortunately, great efforts have been made in the development of therapeutic drugs [3], preventive vaccines [4], and the discovery of other measures beneficial for disease recovery. However, the co-infection of SARS-CoV-2 with other microorganisms enhances the severity and mortality of COVID-19 [5–7]. Deciphering the main alteration in the composition of microorganism in COVID-19 patients is crucial for effective patient management and treatment of SARS-CoV-2.

The co-infecting microorganisms in COVID-19 patients included bacteria [7], fungi [6], and virus [8]. Recent clinical and in silico studies revealed that viral co-infections in COVID-19 primarily involve respiratory viruses such as enterovirus/rhinovirus (hRV), human metapneumovirus (hMPV), and Respiratory Syncytial Virus (RSV) [9]. The most frequently identified co-infected bacterial pathogens include *Streptococcus pneumoniae*, *Staphylococcus aureus, Klebsiella pneumoniae*, *Acinetobacter baumannii*, *Mycoplasma pneumoniae*, *Legionella pneumophila*, and *Chlamydia pneumoniae* [10]. Fungal originated co-infections including pulmonary aspergillosis and candidiasis were reported to aggravate the severity of SARS-CoV-2 infection [11, 12]. COVID-19-associated pulmonary aspergillosis (CAPA) affects approximately 15% of critically ill patients diagnosed with COVID-19 [13]. Invasive candidiasis is rare but associated with considerable mortality in critically ill patients [14]. *Candida albicans* (*C. albicans*) was identified as the most prevalent pathogen, accounting for 70.7% of cases, in hospitalized COVID-19 patients with oropharyngeal candidiasis (OPC) [15]. However, whether the SARS-CoV-2 infection affects the microbiome structure of the respiratory tract is still unknown. Moreover, the promising pathogens need to be clarified to prevent and treat SARS-CoV-2 infection effectively.

By comparing the structure of bacteria and fungi composition of patients with respiratory tract infections in patients with or without COVID-19 infection using 16S ribosomal RNA (16S rRNA) and Internal Transcribed Spacer (ITS) sequencing, we observed that there are no significant differences in bacterial composition between the two groups. Instead, there is a significantly increased abundance of *C. albicans* from respiratory tract samples in COVID-19 patients. *C. albicans* isolated from COVID-19 infection group showed impaired levels of secreted aspartyl proteinases (Sap), which may help *C. albicans* colonization by evading immune surveillance.

## Result

### The clinical analysis of the recruitments

In total, 35 sputum samples were collected from Ren Ji Hospital, Shanghai Jiao Tong University School of Medicine in January 2023. The clinical data and laboratory test results for 35 patients are presented in Table 1. Among the COVID-19 negative group, there were 18 patients with an average age of 75 years, and 15 (83%) of them were male. In the COVID-19 positive group, there were 17 patients with an average age of 78 years, and 14 (82%) of them were male. There were no statistically significant differences in age (Z = -0.777, *P* = 0.437), gender (Z = 0.077, *P* = 0.939), lymphocytes (19.35, Z = -1.242, *P* = 0.225), neutrophils (80.10, Z = -1.398, *P* = 0.164), and monocytes (7.35, Z = -1.934, *P* = 0.055) between the two groups. Of note, the content of procalcitonin (PCT) was significantly higher in the COVID-19 positive group (0.34 ng/mL) compared with the negative group (0.08 ng/mL) (Z = -2.311, *P* = 0.020).

**Table 1:** Clinical analysis of the recruitments

COVID-19	n	Age	Male	Lymphocyte	Neutrophil	Monocyte	Eosinophil
		x̅±s Y/O	n(%)	M (P_25_, P_75_) %	M (P_25_, P_75_) %	M (P_25_, P_75_) %	M (P_25_, P_75_) %
**Negative**	18	75 ± 9	15(83%)	29.40(20.18,40.40)	84.05(77.25,91.78)	5.80(3.63,6.75)	0.00(0.00,0.20)
**Positive**	17	78 ± 11	14(82%)	19.35(7.93,33.65)	80.10(64.08,84.38)	7.35(4.93,12.75)	0.15(0.00,1.00)
**Z-value**		-0.777	0.077	-1.242	-1.397	-1.934	-1.646
**P-value**		0.437^a^	0.939^b^	0.225^a^	0.164^a^	0.055^a^	0.117^a^
**COVID-19**	**n**	**Basophile**	**PCT**	**IL-6**	**CRP**	**D-Dimer**	**FDP**
		M (P_25_, P_75_) %	M (P_25_, P_75_) ng/mL	M (P_25_, P_75_) pg/mL	M (P_25_, P_75_) mg/L	M (P_25_, P_75_) mg/L	M (P_25_, P_75_) µg/mL
**Negative**	18	0.10(0.00,0.13)	0.08(0.63,0.17)	12.38(3.66,19.24)	39.51(11.16,60.72)	1.09(0.50,2.45)	9.50(4.41,16.10)
**Positive**	17	0.20(0.10,0.28)	0.34(0.00,0.79)	15.04(1.62,43.94)	14.29(3.04,24.79)	0.63(0.18,4.45)	3.10(2.10,26.12)
**Z-value**		-1.718	-2.311	-0.154	-1.898	-0.906	-1.399
**P-value**		0.102^a^	0.020^a^	0.910^a^	0.057^a^	0.370^a^	0.165^a^

a, Mann-Whitney U test; b, Chi-square test

### Bacterial composition is similar in the respiratory tract of patients with or without COVID-19 infection

**Fig. 1:**
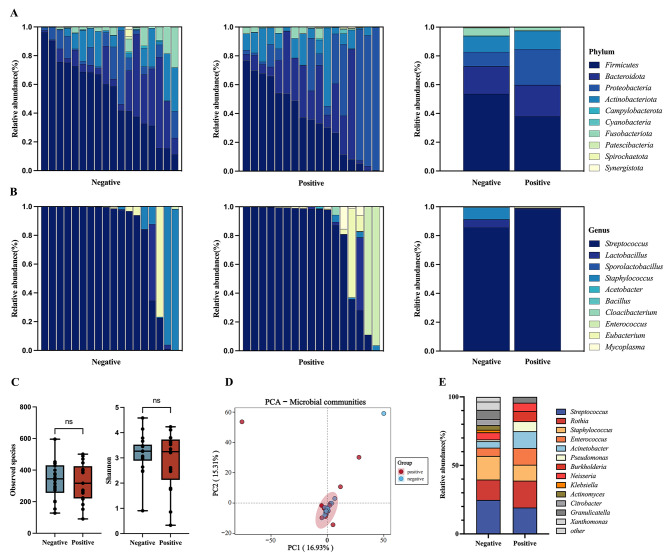
Respiratory tract bacterial composition comparisons between COVID-19 infected and non-infected patients **(A)** Bar chart showing the relative abundance of the top 10 phylum of in the COVID-19 negative and positive groups. **(B)** Bar chart showing the relative abundance of the top 10 genus of *Firmicutes* in the COVID-19 negative and positive groups. **(C)** Alpha diversity analysis. **(D)** Beta diversity analysis. **(E)** Relative abundance of bacterial isolates at the culture level. The statistical significance was measured by unpaired, two-tailed Student’s t test. ns, not significant

The increased contents of PCT suggested a potential bacterial infections in the COVID-19 positive group [16], the bacterial microbiome was first compared between individuals with positive and negative COVID-19 respiratory tract infections utilizing 16S rRNA sequencing. At the phylum level, no significant distinctions in microbial composition were observed between the two groups as *Firmicutes* is the dominant phylum in both groups. Interestingly, there was a reduction in the relative abundance of *Firmicutes* (36.86%) in the COVID-19 positive group compared to the negative group (53.27%) (Fig. 1A). Further analysis at the genus level demonstrated that *Streptococcus* was the prevailing genus in both groups (Fig. 1B), with a prevalence of 46.67% in the negative group and 32.24% in the positive group. There are no differences in the alpha diversity, as determined by the observed species and Shannon indicators (Fig. 1C), and beta diversity through principal component analysis (PCA) (Fig. 1D) between the two groups. In order to validate these findings, a culture-based methodology was utilized, confirming *Streptococcus* as the prevailing genus in both groups (Fig. 1E). Collectively, our results indicate that there are no substantial disparities in the bacterial composition of the upper respiratory tract among individuals post COVID-19 infection.

### The abundance of *Candida* exhibited a significant increase in the upper respiratory tract of the COVID-19 positive patients

**Fig. 2:**
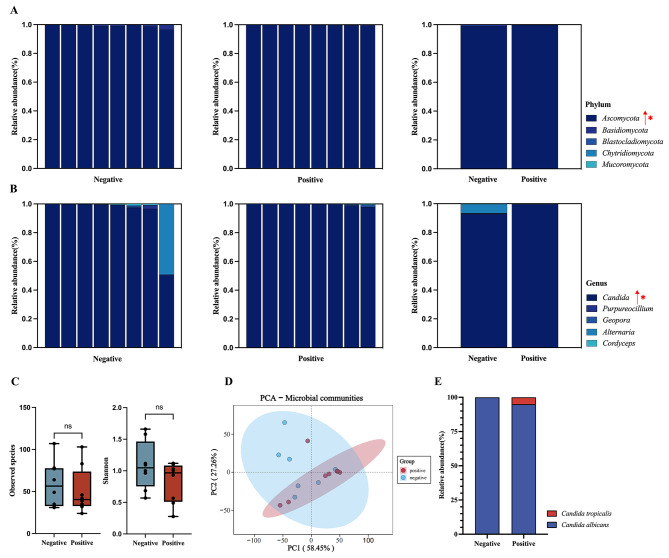
Increased *Candida* abundance in the upper respiratory tract of COVID-19 positive patients **(A)** Bar chart showing the relative abundance of the top 5 phylum in the COVID-19 negative and positive groups. **(B)** Bar chart showing the relative abundance of the top 5 genus in the COVID-19 negative and positive groups. **(C)** Alpha diversity analysis. **(D)** Beta diversity analysis. **(E)** Relative abundance of fungal isolates at the culture level. The statistical significance was measured by Mann-Whitney U test. ns, not significant. *, *P* < 0.05

Fungal infections affect the severity of COVID-19 patients [17], therefore, a detailed analysis of fungal composition was conducted through ITS sequencing. At the phylum level, the microbial structure of the COVID-19 positive group was predominantly characterized by the presence of *Ascomycota* (Fig. 2A), with *Saccharomycetales* at the order level and *Saccharomycetes* at the class level (LDA SCORE [log 10] > 3) (Figure S1). Conversely, the COVID-19 negative group exhibited a predominance of *Fusarium*, *Meyerozyma*, and *Malassezia restricta* at the genus level (|LDA SCORE [log 10]| > 2) (Figure S1). Furthermore, analysis of the top 5 dominant genus showed that *Candida* species is the prevailing fungal genus in both groups (Fig. 2B), and *Candida* species showed a significantly higher abundance in the COVID-19 positive group (Fig. 2B). The analysis of observed species and Shannon indices indicated a declining trend of Alpha diversity of the upper respiratory tract in the COVID-19 positive group (Fig. 2C). Beta diversity analysis demonstrated that the first principal component significantly contributed to the intergroup dissimilarities (Fig. 2D). Finally, culture-based isolation analysis showed that *C. albicans* is the predominant fungi in both groups (Fig. 2E). Moreover, the separation rate of *C. albicans* in the COVID-19 positive group (80/411, 19.46%) surpasses that of the COVID-19 negative group (49/432, 11.34%), which highlights a higher prevalence of *C. albicans* in individuals with COVID-19 infection. Taken together, our data suggested a substantial elevation of *Candida* in the upper respiratory tract in the COVID-19 positive group, with *C. albicans* being the predominant species in the sputum samples of both groups.

### Metabolic pathways in bacterial and fungal colonization patterns in respiratory infections

**Fig. 3:**
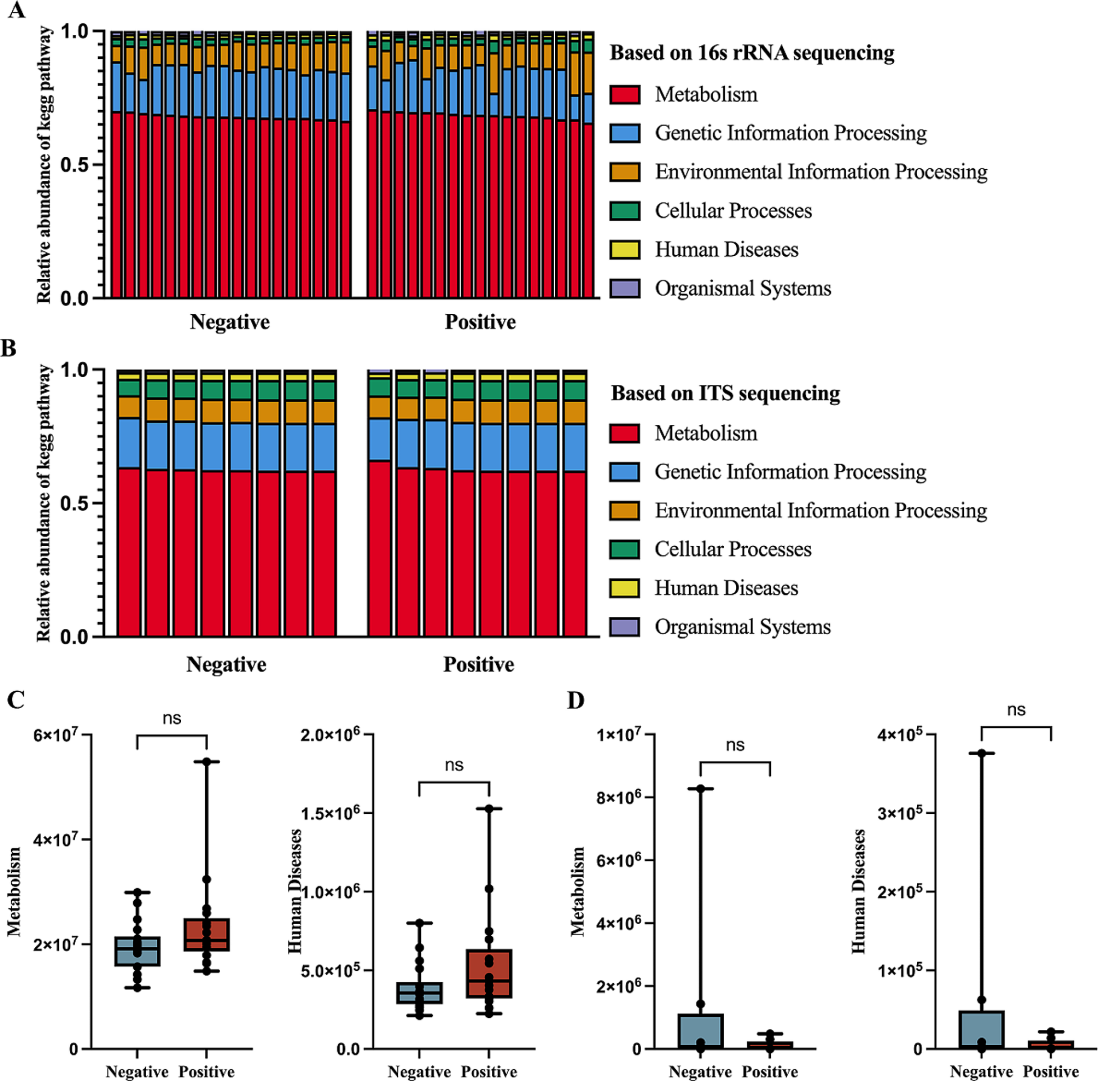
Metabolic pathways of bacterial and fungal colonization in respiratory infections **(A)** KEGG pathway analysis of bacteria. **(B)** KEGG pathway analysis of fungi. **(C)** Comparison of bacterial-related metabolic and disease levels between the two groups. **(D)** Comparison of fungal-related metabolic and disease levels between the two groups. The statistical significance was measured by unpaired, two-tailed Student’s t test. ns, not significant

Based on our comprehensive analysis of 16S rRNA and ITS data, we conducted a meticulous KEGG pathway analysis to investigate the potential metabolic impact exerted by both bacterial and fungal communities between the two groups. Notably, the colonization of specific bacterial species within sputum samples was found to significantly influence the modulation of host cellular metabolic pathways (Fig. 3A&B). However, there are no statistically significant differences observed between the two groups pertaining to these metabolically relevant pathways (Fig. 3C&D). This suggests that the establishment of pathogenic bacteria can affect the metabolic pathways of the host, irrespective of the presence of a COVID-19 infection.

### Comparative expression of secreted aspartyl proteinases and phospholipases in *Candida albicans* isolated from COVID-19 positive and negative groups

*C. albicans* possesses multiple virulence factors, including hyphal formation, surface recognition molecules, phenotypic switching, secretion of extracellular hydrolytic enzymes, adhesion, and tissue penetration ability [18]. Among these factors, the secretion of hydrolytic enzymes plays a crucial role in invading host tissues, with most strains producing high levels of secreted aspartyl proteinases (Sap), phospholipases (Plb), and lipases (Lip) [19]. As shown in Table 2, in the COVID-19 negative group, 35 strains of *C. albicans* showed positive expression for secreted aspartyl proteinases (97.22%), while 8 strains showed positive expression for phospholipases (25.80%). In the COVID-19 positive group, 30 strains exhibited positive expression for secreted aspartyl proteinases (88.24%), and 16 strains showed positive expression for phospholipases (45.71%). We observed that the expression of Sap was significantly lower in the strains isolated from the upper respiratory tract in the COVID-19 positive group compared to those from the COVID-19 negative group (*P* < 0.0001).

**Table 2:** Comparative Expression of Secreted Aspartyl Proteinases (Sap) and Phospholipases (Plb) in *Candida albicans* isolated from COVID-19 positive and negative groups

Type	Group	n	Enzyme expression				P-value*
			High	Medium	Low	None	
**Sap**	Negative	36	26	8	1	1	< 0.0001
	Positive	34	9	8	13	4	
**Plb**	Negative	31	1	4	3	23	0.0760
	Positive	35	9	5	2	19	

*Chi-square test

## Discussion

In this study, we found that there was no significant difference in the bacterial composition of the upper respiratory tract between COVID-19 positive and negative individuals. However, COVID-19 positive patients showed a significant increase in the abundance of *C. albicans*, which displayed a concurrent decrease in the level of secreted aspartyl proteinases compared with the strains isolated from COVID-19 negative patients, suggesting alterations in the microbial composition of the upper respiratory tract.

Changes in the respiratory microbiota have been associated with disease severity [20]. In our study, we did not observe significant differences in bacterial composition using 16S rRNA sequencing. It has been reported that the upper respiratory tract microbiota of COVID-19 positive patients is dominated by Gram-positive *Staphylococcus* and *Corynebacterium* species [21]. Another study indicates that, compared to COVID-negative individuals, the upper respiratory tract microbiota of COVID-positive patients predominantly consists of *Streptococcus* and *Veillonella* [22]. The alterations in microbiota of patients with COVID-19 may be attributed to differences in sampling location (upper or lower respiratory tract), sampling methods, patient severity, disease stage, antibiotic usage, length of ICU stay, and other confounding factors [23]. It is possible that the similar bacterial composition is due to the limitations in sample size in our study.

However, a significant increase in the relative abundance of *C. albicans* in the upper respiratory tract of COVID-19 positive patients was observed by ITS sequencing and culture-based analysis in our study (Fig. 2). The significant threat in the treatment of COVID-19 co-infection with *C. albicans* has been well described. Furthermore, studies have demonstrated a link between *C. albicans* and the development of Long COVID or Post-acute sequelae of COVID-19 (PASC) [24–26]. *C. albicans* is a commensal fungus that asymptomatically colonizes the skin and mucosa of 60% of healthy individuals. However, *C. albicans* has the capacity to transition from a commensal to an invasive state, and this transition is facilitated by host factors such as: (i) disruption of the normal mucosal flora balance; (ii) compromised barrier functions; and (iii) immunosuppression, particularly decreased cellular immune responses [27]. During the course of COVID-19 infection, SARS-CoV-2 exhibits a propensity to target T cells, B cells, and NK cells, resulting in immune system impairment [28]. This immune dysregulation creates a favorable environment for the proliferation of opportunistic pathogens. It has been reported that the incidence of invasive pulmonary aspergillosis, caused by *Aspergillus* species, in COVID-19 patients ranges from 19.6–33.3% [17]. *C. albicans* is an opportunistic fungi that can cause infections in individuals with compromised immune systems [29]. Due to the attenuated immune response of COVID-19 patients, particularly the reduced upregulation of monocyte CD80 expression and the suppressed release of key cytokines such as IL-6, TNF, IL-1a, and IL-1b, this may lead to a decreased ability to clear *C. albicans* in these patients [30].

We observed that the main species of *C. albicans* isolated from the two groups, exhibited differences in secreting extracellular hydrolytic enzymes (Table 2). The main steps of *C. albicans* infection involve adhesion and hyphal formation. The secreted aspartyl protease family (SAP family) contributes to adhesion, while phospholipase (PLB) facilitates the hydrolysis of phospholipids [31]. Heightened expression of Sap correlates with hyphal formation and enhances adhesion and invasion capabilities [32]. The hydrolytic enzyme production is controlled by a diverse group of genes known as the Saps gene family, consisting of at least 10 members [33]. Sap1 and Sap3 are induced by phenotypic switching, Sap4, Sap5 and Sap6 are expressed upon hyphal formation, and Sap1-Sap6 as well as Sap9-Sap10 are involved in adhesion to host cells. Eight Sap (Sap1-Sap6, Sap9 and Sap10) are important for pathogenicity attributes [34]. Furthermore, Sap have been found to potently induce the production of pro-inflammatory cytokines in monocytes [35]. Recent studies have demonstrated that Sap activate NLRP3 inflammasomes, resulting in the triggering of inflammatory immune responses and the recruitment of neutrophils [36]. Moreover, studies have also pointed out that the secreted aspartyl proteases Sap1, Sap2, and Sap3 from *C. albicans* exhibit the ability to hydrolyze and thereby disrupt the functions of human complement components C3b, C4b, and C5. This suggests that these secreted aspartyl proteases may play a role in enhancing *C. albicans*’ resistance to immune system attacks [34].

We observed a noteworthy decrease in the levels of secreted aspartyl proteases in the COVID-19 positive group when compared to the COVID-19 negative group. Regrettably, the specific aspartyl protease responsible for this alteration remains unidentified. We postulate that *C. albicans* may employ a reduction in aspartyl protease levels as a tactic to evade the immune system’s assault. Nonetheless, there exists the possibility that the reduced colonization of aspartyl protease-producing *C. albicans* in COVID-19 patients could stem from impairments in their immune system [37]. Whether other virulence factors, such as biofilm formation or immune evasion molecules contribute to the colonization of *C. albicans* remains a subject worthy of further investigation [27, 38].

Our findings revealed that COVID-19 positive patients showed significantly higher levels of procalcitonin (PCT) compared to the negative group (Table 1). PCT is a biomarker for bacterial infections [39]. However, the bacterial structure was not affected by SARS-CoV-2 infection by 16S rRNA sequencing. Although the abundance of *C. albicans* is increased significantly in the COVID-19 positive group, the connection between PCT with *C. albicans* or SARS-CoV-2 infection remains to be determined. It is possible that the treatment or medication history of the enrollments may affect the biomarkers for the acute infection. To determine the connection between PCT levels and *C. albicans* or SARS-CoV-2 infection, additional population with SARS-CoV-2 infection transition to negative should be recruited. By examining a more diverse population and accounting for variables such as treatment history, we can better understand the potential links between these factors.

There are several limitations in our study. Firstly, we had a low number of samples from both groups, which may limit the generalizability of our findings. Additionally, information regarding comorbidity was missing, which could impact our comprehensive understanding of host-microbe interactions. Furthermore, the 16S rRNA and ITS sequencing data we utilized only pertained to microbial pathways, thus necessitating the use of RNA-seq for further exploration of host pathways. Regarding the 16S rRNA sequencing, we specifically amplified the V1-V3 region, which is more favorable for *Staphylococcus* and may result in missing information about other microbes. To address these limitations, future studies should consider increasing the sample size, improving the collection of comorbidity information, and incorporating more comprehensive host genome sequencing techniques such as RNA-seq, whole genome sequencing (WGS), and targeted sequencing. Additionally, the identification of Sap through Western Blotting (WB) should be included to obtain more comprehensive and accurate results.

## Methods

### Participant enrollment and samples collection

From January 9th to 11th, 2023, a total of 94 sputum samples were collected from patients. After preliminary screening based on clinical diagnosis, we identified 51 patients with respiratory tract infections. Subsequently, through nucleic acid testing for SARS-CoV-2, we further screened 35 patients and divided them into two groups based on their test results: 17 patients who tested positive for the virus were included in the positive group, while 18 patients who tested negative constituted the negative group.

The human samples collection was approved by the ethics committee of Ren Ji Hospital, School of Medicine, Shanghai Jiao Tong University. In addition, the written informed consents were received from all individuals.

### DNA extraction and PCR amplification

Microbial DNA was extracted from sputum samples using the E.Z.N.A.® Soil DNA Kit (Omega Bio-tek, Norcross, GA, U.S.) following the manufacturer’s protocols. For bacterial analysis, the V1-V3 region of the 16S ribosomal RNA gene was amplified by PCR using specific primers for this region (8F:5’- AGAGTTTGATCCTGGCTCAG-3’ and 533R: 5’- TTACCGCGGCTGCTGGCAC-3’). Similarly, for fungal analysis, the ITS1-ITS2 region of the ITS ribosomal RNA gene was amplified by PCR using specific primers (ITS1F: 5’- CTTGGTCATTTAGAGGAAGTAA-3’ and ITS2R: 5’- GCTGCGTTCTTCATCGATGC-3’). The amplified DNA products were then pooled and used to construct an Illumina Pair-End library following Illumina’s genomic DNA library preparation procedure. The amplicon library was subsequently subjected to paired-end sequencing (2*250) on an Illumina MiSeq platform (Shanghai BIOZERON Co., Ltd) according to standard protocols. To ensure data accessibility and availability, the raw sequence reads obtained from the sequencing process were deposited into the NCBI Sequence Read Archive (SRA) database under the designated Accession Number: PRJNA1013128 and PRJNA1013618.

### Data analysis

Figure S2A and S2B showed the Shannon-Wiener index for 16S rRNA and ITS sequencing, which indicates that the sequencing data volume is sufficiently large to reflect the majority of microbial information in the sample. The OTU (Operational Taxonomic Units) were clustered using UPARSE (version 7.1, http://drive5.com/uparse/) with a 97% similarity cutoff. Chimeric sequences were identified and removed using UCHIME [40]. Rarefaction analysis, based on Mothur v.1.21.1 literature, was conducted to assess diversity indices such as Chao, ACE, and Shannon diversity indices [41]. Beta diversity analysis was performed using UniFrac literature to compare the results of principal component analysis (PCA) [42]. The community ecology package R-forge was utilized, and the Vegan 2.0 package was used to generate a PCA figure. For the identification of biomarkers for highly dimensional colonic bacteria, LEfSe (linear discriminant analysis effect size) analysis was conducted following specific literature [43]. The Kruskal-Wallis sum-rank test was employed to examine changes and dissimilarities among classes, followed by LDA (Linear Discriminant Analysis) analysis to determine the effect size of each distinctively abundant taxa, as mentioned in relevant literature [44]. Phylogenetic Investigation of Communities by Reconstruction of Unobserved States (PICRUSt) (http://picrust.github.io/picrust/tutorials/genome_prediction.html) program based on the Kyoto Encyclopedia of Genes and Genomes (KEGG) database was used to predict the functional alteration of microbiota in different samples.

### Isolation and identification of microorganisms

All the sputum samples collected from the recruitment process were mixed with 5 ml of sterile saline. After vortexing, 100 µl of each sample was cultured on sheep blood agar at 37 °C for 24 h to isolate bacteria. At least 24 colonies were selected and subjected to species identification using MALDI-TOF-MS (Bruker Daltonics, Bremen, Germany). For species identification, a small amount of bacteria was spotted onto a steel target plate and then treated with 1 µl of 10% formic acid (Sigma F0507). This mixture was dried for 5 min at 75 °C. Next, 1 µl of MALDI matrix saturated solution (consisting of a-cyano-4-hydroxycinnamic acid from Sigma 70,990 in 50% acetonitrile / 2.5% trifluoroacetic acid) was added to the bacteria. The plate was then analyzed using the MALDI-TOF MS system. The spectrum was obtained in linear positive-ion mode within the range of 2000 to 20,000 Da. Each spot was measured manually at five different positions, with 1000 laser shots at 25 Hz in groups of 40 shots. The acquired spectra were analyzed using MALDI Bruker Biotyper 3.0 software and library (Bruker Daltonics).

### Reagent formulation

Bovine serum albumin plate: A 10% solution of bovine serum albumin was prepared by dissolving 20.0 g of glucose, 0.2 g of yeast extract powder, 1.0 g of KH2PO4, and 20.0 g of agar in 1 L of distilled water. The pH was adjusted to 5.6 ± 0.2. The bovine serum albumin solution was added to a final concentration of 1%. The plates were poured after thorough mixing.

Egg yolk agar medium: A medium was prepared by dissolving 40.0 g of glucose, 10.0 g of peptone, 63.6 g of NaCl, 0.6 g of CaCl2, and 15.0 g of agar in 1 L of distilled water. The pH was adjusted to 5.6 ± 0.2. was After autoclaving, the medium was cooled to 50 °C, and mixed with 100mL of egg yolk emulsion, and the plates were poured after thorough mixing.

### Detection of secretory hydrolase activity

The suspension for pathogens were adjusted with a turbidity of 0.5 McFarland in PBS buffer. After diluted at a ratio of 1:200, the pathogen was spotted on the agar plates. The plates were incubated at 37 °C incubator after drying. The diameter of the colonies and the diameter of the halo were measured after incubating for 72 h. The Pz value was calculated using the formula: Pz = Colony diameter / (Colony diameter + Halo diameter). A higher Pz value indicates less secretion of hydrolases. Pz ≤ 0.59 is considered high expression of hydrolases; Pz values between 0.6 and 0.79 indicate moderate expression of hydrolases, Pz values between 0.8 and 1 indicate low-level expression of hydrolases, and Pz = 1 indicates no expression of hydrolases.

### Statistical analyses

Statistical analysis was conducted using IBM SPSS 27 and GraphPad Prism 9. For quantitative data such as age, PCT, IL-6, independent-sample t-tests, Mann-Whitney tests, and Kruskal-Wallis tests were employed for statistical analysis. For categorical data such as gender, Chi-Square tests were used. A significance level of *P* < 0.05 was considered statistically significant.

### Electronic supplementary material

Below is the link to the electronic supplementary material.

Supplementary Material 1

## Data Availability

All data generated or analyzed during this study are included in this published article. The datasets generated and analyzed during the current study are available in the NCBI repository, the IDs of 16S rRNA and ITS sequencing are PRJNA1013128 and PRJNA1013618.
