# Supplementary material for: The characteristics of microbiome in the upper respiratory tract of COVID-19 patients
Source: BMC Microbiol. 2024 Apr 24;24:138. doi: 10.1186/s12866-024-03281-w (PMC11040800; doi:10.1186/s12866-024-03281-w)
Supplement: Supplementary file 1 — Supplementary Material 1 [file 12866_2024_3281_MOESM1_ESM.docx]

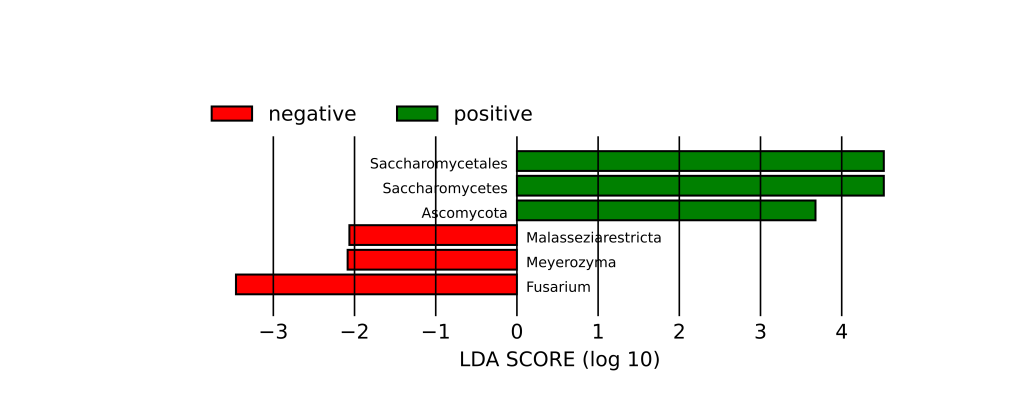


**Figure S1 The relative abundance of fungal genera at the inter-group level using LAD analysis.** At the phylum level, the microbial structure of the COVID-19 positive group was predominantly characterized by the presence of *Ascomycota*, with *Saccharomycetales* at the order level and *Saccharomycetes* at the class level (LDA SCORE [log 10] > 3). Conversely, the COVID-19 negative group exhibited a predominance of *Fusarium*, *Meyerozyma*, and *Malassezia restricta* at the genus level (|LDA SCORE [log 10]| > 2)


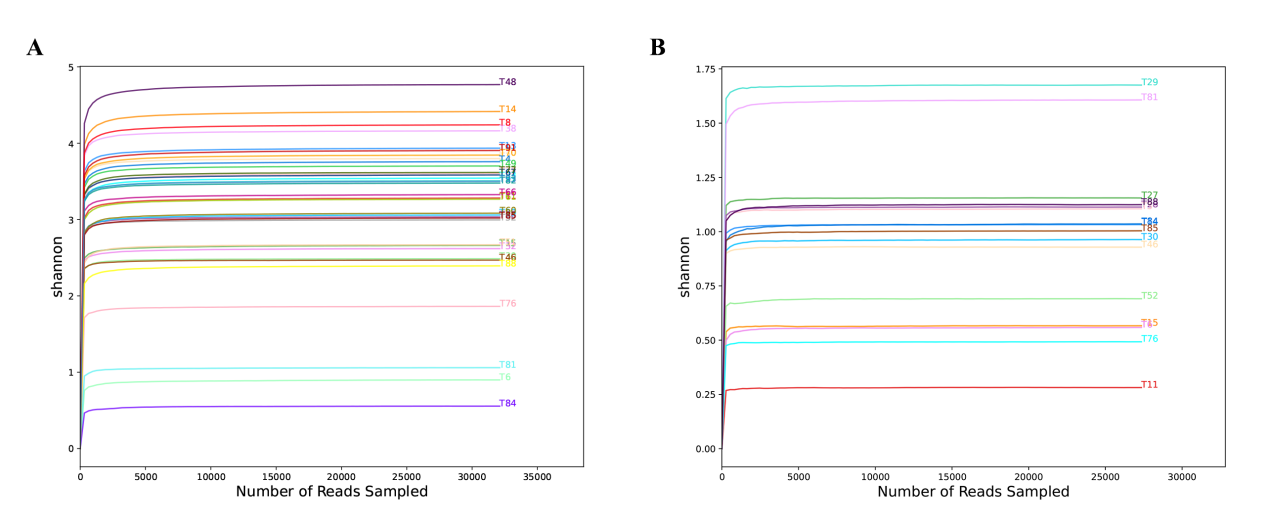


**Figure S2** **The Shannon-Wiener index**. **(A)** 16S rRNA for bacteria. **(B)** ITS for fungi. When the curve tends to flatten, it indicates that the sequencing data volume is sufficiently large to reflect the majority of microbial information in the sample.
